# Asiatic Acid Prevents the Deleterious Effects of Valproic Acid on Cognition and Hippocampal Cell Proliferation and Survival

**DOI:** 10.3390/nu8050303

**Published:** 2016-05-18

**Authors:** Jariya Umka Welbat, Apiwat Sirichoat, Wunnee Chaijaroonkhanarak, Parichat Prachaney, Wanassanun Pannangrong, Poungrat Pakdeechote, Bungorn Sripanidkulchai, Peter Wigmore

**Affiliations:** 1 Department of Anatomy, Faculty of Medicine, Khon Kaen University, Khon Kaen 40002, Thailand; dek_saba@hotmail.com (A.S.); cwunnee@kku.ac.th (W.C.); parpra@kku.ac.th (P.P.); wankun@kku.ac.th (W.P.); 2 Center for Research and Development of Herbal Health Products, Khon Kaen University, Khon Kaen 40002, Thailand; bungorn@kku.ac.th; 3 Neuroscience Research and Development Group, Khon Kaen University, Khon Kaen 40002, Thailand; 4 Department of Physiology, Faculty of Medicine, Khon Kaen University, Khon Kaen 40002, Thailand; ppoung@kku.ac.th; 5 School of Life Sciences, Medical School, Queen’s Medical Centre, Nottingham University, Nottingham NG7 2UH, UK; peter.wigmore@nottingham.ac.uk

**Keywords:** valproic acid, Asiatic acid, neurogenesis, spatial memory

## Abstract

Valproic acid (VPA) is commonly prescribed as an anticonvulsant and mood stabilizer used in the treatment of epilepsy and bipolar disorder. A recent study has demonstrated that VPA reduces histone deacetylase (HDAC) activity, an action which is believed to contribute to the effects of VPA on neural stem cell proliferation and differentiation which may explain the cognitive impairments produced in rodents and patients. Asiatic acid is a triterpenoid derived from the medicinal plant *Centella asiatica*. Our previous study has shown that Asiatic acid improves working spatial memory and increases cell proliferation in the sub granular zone of the hippocampal dentate gyrus. In the present study we investigate the effects of Asiatic acid in preventing the memory and cellular effects of VPA. Male Spraque-Dawley rats were orally administered Asiatic acid (30 mg/kg/day) for 28 days, while VPA-treated animals received injections of VPA (300 mg/kg) twice a day from Day 15 to Day 28 for 14 days. Spatial memory was determined using the novel object location (NOL) test and hippocampal cell proliferation and survival was quantified by immuostaining for Ki-67 and Bromodeoxyuridine (BrdU), respectively. The results showed that VPA-treated animals were unable to discriminate between objects in familiar and novel locations. Moreover, VPA significantly reduced numbers of Ki-67 and BrdU positive cells. These results indicate that VPA treatment caused impairments of spatial working memory, cell proliferation and survival in the subgranular zone (SGZ) of the hippocampal dentate gyrus (DG). However, these abnormalities were restored to control levels by co-treatment with Asiatic acid. These data demonstrate that Asiatic acid could prevent the spatial memory and neurogenesis impairments caused by VPA.

## 1. Introduction

Valproic acid (VPA) is commonly prescribed as an anticonvulsant and mood stabilizer and is used in the treatment of epilepsy and bipolar disorder [1,2]. It is also used to treat other disorders including migraine headaches and schizophrenia [1,3]. VPA treatment induces neuronal damage and contributes to the aberrant neurogenesis associated with epileptic activity in adult rodents [4,5]. A recent study has demonstrated that the effects of VPA in inhibiting histone deacetylase (HDAC) activity, which produces epigenetic changes in gene expression, could be an explanation for the changes in cell proliferation and differentiation produced by VPA treatment [6]. A high dose of VPA reduces HDAC activity and HDAC-mediated responses, effects that are believed to contribute to the impact of VPA on neuronal differentiation in both normal and epileptic adult rodent brain [7]. Animals given VPA 300 mg/kg twice daily showed a reduction in cell proliferation in the subgranular zone (SGZ) of hippocampal dentate gyrus (DG), which was associated with spatial memory deficits [8]. Patients Undergoing VPA therapy for a period of time often show changes in mood, cognition and behavior. Previous studies have shown that VPA can obviously develop cognitive dysfunction in epileptic patients [9,10,11]. In addition to these effects, VPA has also been found to induce oxidative stress and cause a variety of toxicities [12,13].

The SGZ of the hippocampal DG is one of two main neurogenic regions in the adult brain [14,15]. Neural progenitor cells (NPCs) in the SGZ proliferate and differentiate into dentate gyrus granule cells. Following a period of maturation, these newly generated cells integrate into the pre-existing neural circuitry where they functionally contribute to the processes of learning and memory [16,17,18]. Adult neurogenesis has been found to be required for certain types of memory that are associated with the hippocampus in particular spatial memory and pattern separation (REF). Anti-proliferative agents have been shown to produce a decrease in hippocampal neurogenesis, which is associated with hippocampal specific memory deficits [19,20]. In animal models, the number of adult newborn neurons increases in the hippocampus when animals perform hippocampal-dependent learning tasks [21]. These studies indicate that new neurons in the hippocampus are required for and influenced by the process of memory formation [21,22,23]. Other studies have examined the number of new neurons in relation to task performance and found that generally, those animals with the fewest new neurons perform the poorest while those with the most new neurons perform the best [8,24,25]. 

Asiatic acid is a triterpenoid derived from the medicinal plant *Centella asiatica* (*C. asiatica*) [26,27], which is an aromatically weak, slender and creeping perennial herb used by diverse ancient cultures in Thailand and other Asian countries [27,28,29]. Asiatic acid has been reported to have biological effects including acting as an antioxidant as well as an anti-inflammatory [30,31]. It can cross the blood–brain barrier and has shown neuroprotective effects on cells in culture. These results are associated with its antioxidant effects [32,33,34,35]. Asiatic acid has been shown to improve learning and memory in an animal model an effect that was correlated with an increase in hippocampal neurogenesis [29,35,36]. Furthermore, it also has the ability to enhance hippocampal CA3 neuronal dendritic arborization in rats. Increasing the dendritic length of the hippocampal CA3 neurons may result in alterations in synaptic connectivity [26,37,38], which may improve learning and memory. 

The present study uses an animal model to investigate the preventive effects of Asiatic acid on the cognitive and cellular effects of VPA. Working spatial memory was tested using the novel object location (NOL) test and cell proliferation and the survival of newborn hippocampal neurons were examined using Ki-67 and BrdU immunofluorescence staining, respectively. 

## 2. Materials and Methods

### 2.1. Animals

Male Spraque-Dawley rats weighing between 180 and 200 g were used. The experimental protocol was approved by the Khon Kaen University Ethics Committee in Animal Research (project number AEKKU 30/2556). Animals were maintained under standard laboratory conditions with 25 °C and 12 h dark/light cycle and allowed to have adequate food and water. After 7 days of habituation, the animals were randomly divided into 4 groups (10 animals in each group): control, Asiatic acid, VPA, and Asiatic acid plus VPA groups. 

### 2.2. Drugs Administration

Animals were orally administered Asiatic acid (30 mg/kg/day, dissolved in propylene glycol, Faces Biochemical Co., Ltd., Wuhan, China) via gavage tube in a volume of 1 mL/kg for 28 days, while VPA-treated animals received intraperitoneal (i.p.) injection with VPA (300 mg/kg, dissolved in 0.9% normal saline at a volume of 1 mL/kg, Sigma-Aldrich, Inc., St. Louis, MO, USA), twice a day, on Day 15 to Day 28 for 14 days. All animals received i.p. injections of Bromodeoxyuridine (BrdU) (100 mg/kg, Sigma Aldrich, Saint Louis, MO, USA) on the first day of drug treatment at a volume of 5 mL/kg, once a day for 3 days. 

### 2.3. Novel Object Location (NOL) Test

The novel object location (NOL) test was used to determine spatial working memory after the drug administration. This was a modification of a previous method [8,39]. The apparatus consisted of an arena (a semi-transparent plastic box, dimensions 36-cm wide × 50-cm long × 30-cm high) and plastic bottles were filled with water to weight them down. Experiments were conducted at an illumination of 350–400 Lux and recorded by VDO camcorder (Version-052, KER Crown computer Co., Ltd., Bangkok, Thailand).

The NOL test was carried out 3 days after treatment ended. One day prior to testing, animals were habituated for 30 min by allowing them to freely explore an open-field arena in the absence of objects. The task procedure composes of a familiarization and choice trials with 15 min inter-trial interval. In the familiarization trial, two identical objects were placed in separate corners of the arena and each animal was allowed to explore the objects for 3 min. After returning animals to their home cages for 15 min, animals were placed back to the arena for 3 min. In this trial, one object remained in the same or familiar location while the other object was moved to a new (novel) location. Exploring time was scored when the animal directed its nose at a distance less than 2 cm from the object [39,40]. The preference index was defined as time spent exploring the object in the novel location in the choice trial as a percentage when compared to 50% chance [8].

### 2.4. Immunohistochemistry 

The day after the NOL test, animals were put down by rapid stunning followed by decapitation. Brains were cryoprotected in a 30% sucrose solution for 3 h at 4 °C and then embedded in Optimal Cutting Temperature (OCT) compound (Sakura Finetek, Torrance, CA, USA). They were snap-frozen in liquid nitrogen-cooled isopentane and stored at −80 °C prior to section. Ki-67 was used to quantify cell proliferation in hippocampal dentate gyrus. Frozen brains were serially sectioned (20 µm) in the coronal plane from Bregma point −2.3 to −6.3 mm to include the entire dentate gyrus using a cryostat (Cryostat Series HM 550 Microm international, A.S. Science Co., Ltd., Walldorf, Germany) and mounted on 3-aminopropyl-methoxysilane (APS) coated slides. Sections ware fixed in 0.5% paraformaldehyde (pH 7.4) for 3 min and then sections were incubated with primary antibody monoclonal mouse Ki-67 (1:150, Vector Laboratory, Inc., Burlingame, CA, USA) at room temperature for 1 h. Sections were then incubated with secondary antibody Alexa 488 rabbit anti mouse IgG (1:300, Invitrogen, Carlsbad, CA, USA) for 40 min and counter stained with propidium iodide (PI) (1:6000, Sigma Aldrich, Inc., St. Louis, MO, USA) for 30 s and mounted in glycerol. 

Cell survival was determined by immunostaining for BrdU. Frozen brains were fixed in 4% paraformaldehyde and serially sectioned (40 µm) in the coronal plane using a frozen microtome (Walldorf, Germany). Sections were incubated with 1N hydrochloric acid (HCl) for 10 min on ice to denature the DNA followed by 10 min incubation with 2 N HCl at room temperature before moving them to an incubator for 20 min at 37 °C. Immediately after the acid wash 0.1 M borate buffer was used to neutralize the HCl for 12 min and then sections were incubated and blocked with 5% normal goat serum in PBS containing 0.1% triton X-100 and 1 M Glycine for 1 h. After washing, sections were incubated with primary antibody, polyclonal sheep anti BrdU (1:100, Abcam, Cambridge, UK) in blocking solution overnight. After that, sections were incubated with secondary antibody anti sheep 488 Alexa fluor (1:300, Invitrogen, Carlsbad, CA, USA) in blocking solution for 60 min and counter stained with Propidium iodide (PI) (1:300) and mounted in glycerol.

All sections were viewed and quantified at X40 on a Nikon ECLIPSE 80i fluorescence microscope with NIS-Element AR 3.2 software. Ki-67 and BrdU positive cells which were co-localized with PI nuclear staining and only cells within 3 cell diameters of the inner edge of both blades of the dentate gyrus were scored. A systemic random sampling method [41] was used to choose every 15th section throughout the length of the dentate gyrus (over all 9 sections). The number of Ki-67 and BrdU positive cells in each hippocampus was produced by combining cell counts per section for the whole dentate gyrus and multiplying by 15. 

### 2.5. Statistical Analysis

Statistical analysis was calculated using GraphPad Prism (V. 5.0; GraphPad Software Inc., San Diego, CA, USA), SPSS (V 17.0; SPSS Inc., Chicago, IL, USA) and expressed as mean ± SEM and significance was regarded as *p* < 0.05. The Student *t*-test and one-way ANOVA were used to analyze data. When ANOVA was significant least significant difference (LSD) *post hoc* test was performed. 

## 3. Results

### 3.1. Asiatic Acid Prevents the Spatial Working Memory Deficits Caused by VPA

**Figure 1:**
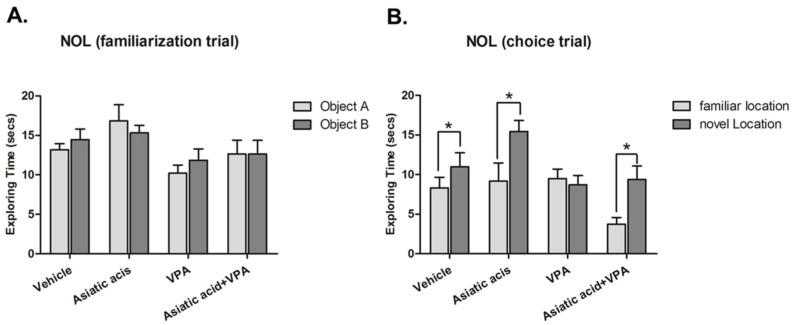
Mean exploration times of the animals exploring each object during the familiarization (**A**) and choice (**B**) trials of the novel object location test after treatment. There were no significant differences in the exploration times of either object for any group in the familiarization (* *p* > 0.05). In the choice trial, vehicle, Asiatic and Valproic acid (VPA) plus Asiatic acid groups spent a significantly longer time exploring the object in the novel location compared with the familiar location (*p* < 0.05), whereas the VPA group failed to discriminate between objects (*p* > 0.05).

The NOL test was used to assess spatial working memory three days after the end of drug administration. This period was used to avoid confounding issues associated with the acute effects of the drugs. In the familiarization trial, animals in all groups showed no significant difference in exploratory time between the objects in the arena (*p* < 0.05, paired Student *t*-test, Figure 1A) indicating no preference for either object in the two locations prior to the choice trial. Fifteen minutes later, one object was moved to a new location and animals were tested for their ability to discriminate between the object in the familiar location (FL) and the object moved to a novel location (NL). During the choice trial, as expected, animals in the vehicle and Asiatic acid groups spent significantly more time attending the object in the novel location (mean ± SEM; FL, vehicle: 6.270 ± 1.172 s, Asiatic acid: 9.181 ± 2.284 s; NL, vehicle: 12.250 ± 2.262 s, Asiatic acid: 15.450 ± 1.381 s, *p* < 0.05, paired Student *t*-test, Figure 1B). This indicated that these animals had no impairments in spatial working memory. A one-way ANOVA analysis with LSD *post hoc* test revealed that animals receiving Asiatic acid spent significantly more time on the object in the novel locations in comparison to each other group (F(3, 37) = 4.074; *p* = 0.014). In contrast, the VPA treated group was impaired in their ability to discriminate between objects in familiar and novel locations resulting in no significant difference in the time spent on these two objects (mean ± SEM; FL: 9.494 ± 1.190 s, NL: 8.712 ± 1.171 s, *p* > 0.05). Animals administered both VPA and Asiatic acid, however, spent significantly more time on the object in the novel location compared with that in the familiar location (mean ± SEM; FL: 3.718 ± 0.857 s, NL: 9.399 ± 1.694 s, *p* < 0.05). This group behaved similarly to control and Asiatic acid alone treated animals and did not show spatial memory deficits exhibited by the VPA group.

**Figure 2:**
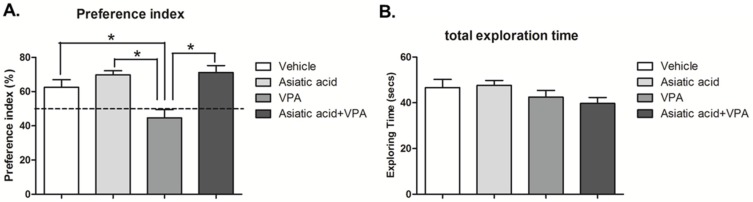
The preference index (PI) showed a significant difference from 50% chance in vehicle, Asiatic acid and VPA plus Asiatic acid groups ((**A**); * *p* < 0.05) while the VPA group showed no significant difference from chance. Additionally, PI in animals in vehicle, Asiatic acid and VPA plus Asiatic acid groups were significantly higher than VPA group (*p* < 0.05). The total exploration time of familiarization and choice trial combined was not significantly different among groups ((**B**); *p* > 0.05).

The exploration times of familiar and novel locations in the choice trial were converted into a preference index (PI). PI was calculated by expressing time spent exploring the object in the novel location as a percentage compared to 50% chance. The data showed that the animals in vehicle, Asiatic acid and VPA plus Asiatic acid treated groups were significantly different from 50% chance (*p* < 0.05; one-sample *t* test, Figure 2A), demonstrating a normal ability in remembering the location of objects and expressing greater interest in objects in novel locations. In contrast, the PI of the VPA group was not different from 50%, indicating spatial working memory deficits. One-way ANOVA with LSD *post-hoc* test confirmed that the PI of vehicle, Asiatic acid and VPA plus Asiatic acid animals were significantly higher than the VPA-treated group (F(3, 29) = 8.851; *p* = 0.003, Figure 2A). The total exploration times showed no significant difference among groups, indicating that animals did not have impaired locomotor ability during the performance of the task (F(3, 32) = 1.500; *p* > 0.05, one-way ANOVA, LSD *post-hoc* test, Figure 2B). 

### 3.2. Asiatic Acid Prevents the Reduction in Cell Proliferation in the SGZ Caused by VPA 

**Figure 3:**
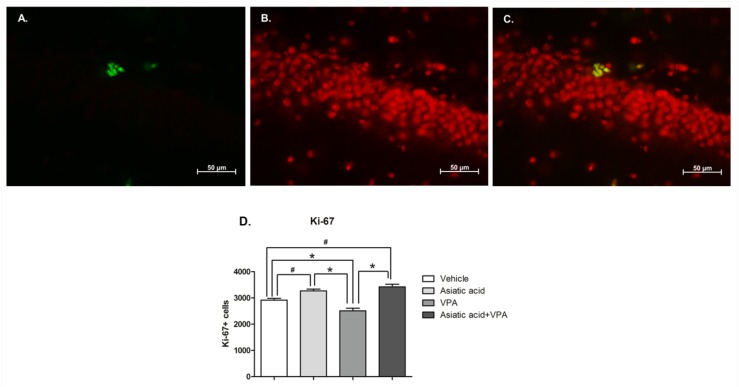
The number of Ki-67 positive cells in the SGZ of the hippocampal dentate gyrus. Ki-67 positive cells were stained in the SGZ of the dentate gyrus (green; (**A**)). All nuclei were counterstained with propidium iodide (red; (**B**) and figure merged (**C**)). The number of proliferating cells in animals receiving only VPA was significantly lower (* *p* < 0.05) than vehicle, Asiatic acid and Asiatic acid plus VPA groups, while Asiatic acid and Asiatic acid plus VPA groups were significantly higher when compared to the vehicle group (# *p* < 0.05). One-way ANOVA with LSD *post hoc* test was used to compare between all groups (**D**).

Ki-67 immunostaining was used to quantify the numbers of proliferating cells in the SGZ of the hippocampal dentate gyrus at the end of the experiment. A one-way ANOVA analysis with a LSD *post hoc* test showed that animals treated with VPA alone had a significantly decreased number of Ki-67 positive cells (*p* < 0.05). Treatment with Asiatic acid alone significantly increased the number of Ki-67 positive cells compared with controls (mean ± SEM; vehicle: 2918 ± 64.31 cells, Asiatic acid: 3270 ± 64.58 cells, VPA: 2513 ± 100.60 cells, and VPA plus Asiatic acid treated groups: 3420 ± 102.10 cells F(3, 20) = 22.65, *p* < 0.001, Figure 3). Interestingly, animals treated with both VPA and Asiatic acid showed no significant difference in Ki-67 positive cell numbers from the vehicle treated control group (*p* > 0.05). This demonstrates that Asiatic acid had prevented the reduction in cell proliferation in the SGZ caused by VPA on its own.

### 3.3. Asiatic Acid Prevents the Reduction in Cell Survival in SGZ Caused by VPA

**Figure 4:**
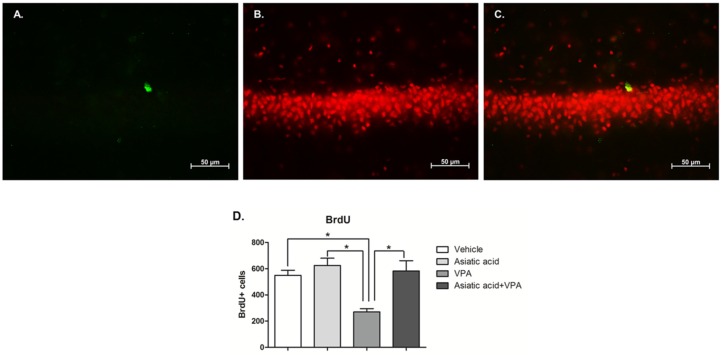
The number of BrdU positive cells in the SGZ of the hippocampal dentate gyrus. BrdU positive cells were stained in the SGZ of the dentate gyrus (green; (**A**)). Nuclei were counterstained with propidium iodide (red; (**B**) and figure merged (**C**)). BrdU positive cell number in VPA-treated group was significantly lower than vehicle, Asiatic acid and Asiatic acid plus VPA groups (* *p* < 0.05). One-way ANOVA with LSD *post hoc* test was used to compare between all groups (**D**).

At the end of the experiment, BrdU-positive cells were counted in the dentate gyrus and SGZ to quantify cell survival. There were significantly fewer BrdU-positive cells in animals treated with VPA when compared with vehicle, Asiatic and VPA plus Asiatic acid (mean ± SEM; vehicle: 549.30 ± 38.85 cells, Asiatic acid: 625.30 ± 34.40 cells, VPA: 270.70 ± 100.24.04 cells and VPA plus Asiatic acid treated groups: 582.70 ± 78.23 cells F(3, 20) = 9.197, *p* < 0.001, one-way ANOVA, LSD *post-hoc* test, Figure 4). These results indicate that VPA treatment reduced cell survival in the SGZ over the course of the experiment. In contrast, animals treated with both VPA and Asiatic acid showed no significant difference in the BrdU-positive cell numbers from vehicle-treated animals (*p* > 0.05). These results suggest that Asiatic acid can protect against the decrease in the survival of proliferating cells found after VPA treatment.

## 4. Discussion

The goal of this study was to understand the impact of VPA and Asiatic acid on spatial memory and hippocampal neurogenesis. Specifically, we wanted to determine whether co treatment with Asiatic acid would be beneficial to VPA exposed animals which can suffer from reduced hippocampal neurogenesis and spatial memory. VPA is an anticonvulsant medication that has been used to effectively control various types of seizure disorders [1,3]. VPA exposure alone has previously been shown to produce cognitive impairments [8]. In the present study, spatial memory was tested using the NOL test, which is hippocampal dependent [8,42]. It relies on the spontaneous preference animals have for objects in novel locations and does not require positive or negative reinforcements [39,43]. Animals with hippocampal abnormalities or damage have been shown to have a poorer performance on this test [8,40,44]. Our results showed that the animals receiving only Asiatic acid were able to discriminate between objects placed in the familiar and novel locations significantly better than vehicle treated controls. This confirms an earlier report that showed that Asiatic acid treatment can improve spatial memory [36]. In contrast, VPA treated animals were impaired in their discrimination between objects in familiar and novel locations, indicating that VPA treatment caused a deficit in spatial working memory [8]. Animals co-administered with both VPA and Asiatic acid showed a significant preference for the object in the novel location compared with that in the familiar location. This result demonstrates that Asiatic acid has a protective effect and prevents spatial memory impairments found in animals exposed to VPA. 

The proliferation and survival of dividing cells can be quantified by immunostaining for Ki-67 and BrdU, respectively. Ki-67 is differentially and endogenously expressed in different phases of the cell cycle [45]. Ki-67 is expressed in dividing cells at all stages of mitotis but is not found in G_0_ [46,47]. BrdU is incorporated into newly formed DNA during S phase of the cell cycle and remains in the nucleus after the completion of cell division [48]. The results of the present study show that VPA significantly reduced the numbers of Ki-67 and BrdU positive cells showing that VPA treatment reduced cell proliferation and survival of dividing cells in the SGZ of the hippocampal dentate gyrus. Recent studies have demonstrated that the VPA inhibits the action HDAC enzymes resulting in expression of growth arrest genes including p21 and apoptosis [6,49]. These effects have been demonstrated on embryonic neural stem cells where they produce brain malformations and abnormal cell migration into the dentate gyrus [50]. VPA exposure has been demonstrated to produce mild cognitive impairments in patients taking this drug for epilepsy or other psychiatric conditions. Moreover, patients experienced long term VPA therapy show slow thinking, cognitive difficulties and problems with motor behavior and display impairments of memory [9,51,52]. VPA has been demonstrated, in the present study and previous publications, to produce a hippocampal specific spatial memory decline, which is correlated with a decrease in hippocampal neurogenesis. This makes the present animal model suitable for use in testing substances for protection from VPA induced cognitive decline. 

Previous reports have shown that Asiatic acid can cross the blood–brain barrier and also has antioxidant activities, which protects against neuronal damage in rat cortical cell primary culture [27,35]. Administration of Asiatic acid significantly restores oxidative stress marker [35] along with an improvement of antioxidant activities [34]. Adverse effects during VPA therapy in patients have been associated with reductions in antioxidant enzymes [12,13]. Decreases of antioxidant enzymes can decrease cell proliferation and survival of neural stem cells [53,54]. Therefore, co-treatment of Asiatic acid and VPA could restore cell proliferation and cell survival in the SGZ of the hippocampus by improving antioxidant pathway. Additionally previous studies have found that Asiatic acid can improve learning and memory in an animal model via an increase of hippocampal neurogenesis [29,36]. The present study demonstrated that co-administration of Asiatic acid with VPA maintained levels of cell proliferation and the survival of dividing cells at control levels. Furthermore, animals treated only with Asiatic acid had significantly higher levels of proliferation than the control group, a result in agreement with our previous study [36]. These findings confirm the neuroprotective effect of Asiatic acid previously reported in cortical cell culture where Asiatic acid was able to rescue primary cortical neuronal cells from C_2_-ceramides induced cell death and against beta-amyloid neurotoxicity when tested on B103 cell cultures and hippocampal slices [27,55,56]. 

In summary, we have shown that administration of Asiatic acid is beneficial in preventing the spatial working memory deficit and cell proliferation and survival stimulated reduction produced by VPA. Therefore, Asiatic acid might be useful in preventing memory deficit in patients taking VPA.
